# Poly(l-Lactide) Liquid Crystals with Tailor-Made Properties Toward a Specific Nematic Mesophase Texture

**DOI:** 10.1021/acssuschemeng.1c08282

**Published:** 2022-03-02

**Authors:** Henryk Janeczek, Khadar Duale, Wanda Sikorska, Marcin Godzierz, Aleksandra Kordyka, Andrzej Marcinkowski, Anna Hercog, Marta Musioł, Marek Kowalczuk, Darinka Christova, Joanna Rydz

**Affiliations:** † Centre of Polymer and Carbon Materials, Polish Academy of Sciences, M. Curie-Skłodowska 34, 41-800 Zabrze, Poland; ‡ School of Science, Faculty of Science and Engineering, University of Wolverhampton, Wulfruna St., Wolverhampton WV1 1LY, U.K.; § Institute of Polymers, Bulgarian Academy of Sciences, Akad. Georgi Bonchev Str., Bl. 103A, 1113 Sofia, Bulgaria

**Keywords:** poly(l-lactide), thermotropicity, liquid crystal, chiral nematic, nematic texture, blue phase

## Abstract

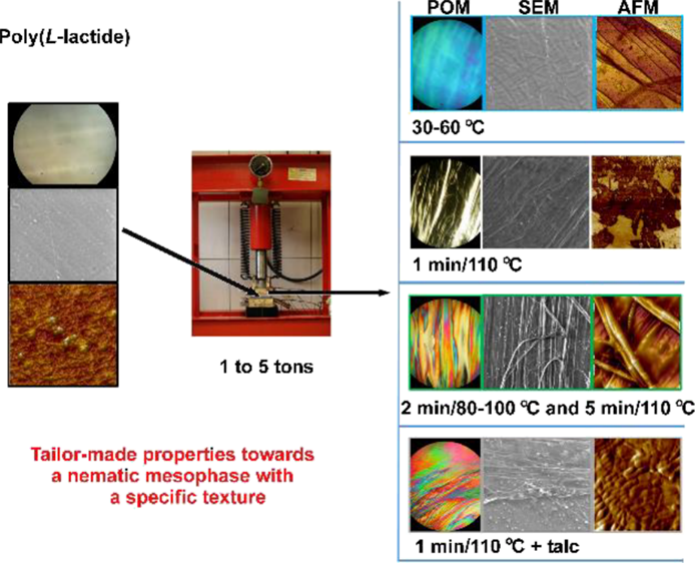

This paper presents the liquid crystal (LC) properties of poly(l-lactide) (PLLA). Mesophase behavior is investigated using polarized optical microscopy, X-ray diffraction, and differential scanning calorimetry. The performed analyses confirm that pressed PLLA films exhibit the unique capability of self-assembling into a nematic mesophase under the influence of mechanical pressure, temperature, and time. It was originally demonstrated that the chiral nematic mesophase can be obtained by introducing fine powders into the polymer. Based on the research conducted, it was proved that the pressed PLLA films have a chiral nematic mesophase with a nematic-to-isotropic phase transition and a large mesophase stability range overlapping the temperature of the human body, which can persist for years at ambient temperature. The obtained films show tailor-made properties toward a nematic mesophase with a specific texture, including colored planar texture of the chiral nematic mesophase and blue-phase (BP) LC texture. The BP, described for the first time in plain PLLA, occurred over a wider than usual temperature range of stability between isotropic and chiral nematic thermotropic phases (Δ*T* ≈ 9 °C), which is an advantage of the obtained polymer material, in addition to ease of preparation. This opens up new prospects for advanced photonic green applications.

## Abstract 2

Poly(l-lactide) pressed films show tailor-made properties toward a nematic mesophase with a specific texture, including blue-phase liquid crystal texture.

## Introduction

Materials in which phase transitions are observed as a function of temperature and that form the mesophase (exhibiting both some of the typical properties of the liquid regarding their mobility at room temperature (RT) and some properties of a crystalline material) are liquid crystals (LCs). LCs are a state of matter that is thermodynamically placed between an isotropic liquid and a three-dimensionally ordered solid. Phase transitions take place at certain temperatures, starting with the breaking of the crystalline order of the solid, causing oscillation or rapid rotation about a given axis (usually the long axis of the molecules). Then the long-range positional order is lost (smectic mesophase, Sm), showing only orientational and short-range positional order within the diffused layers. The local packing order is then destroyed, except for the long orientational order of the long axes (phase director) to produce the nematic (N) mesophase. Ultimately, all order is lost, creating an isotropic liquid. Some compounds pass from the crystalline to the nematic phase, omitting smectic phases.^1−3^ LCs are formed either by heating (thermotropic LCs) or in the presence of solvents (lyotropic LCs).^4,5^ Depending on the spatial orientational and positional order of the molecules, thermotropic LCs can be divided into nematic, chiral nematic (cholesteric), and smectic. The N mesophase, one of the most common and important mesophases in applications, is characterized by a high degree of long-range orientational order, but no translational order, making it the least ordered mesophase of the LCs. Molecules in the N mesophase arrange their long axes in parallel, defining the so-called director. The Sm mesophase usually occurs at lower temperatures than the N mesophase, has a further degree of ordering, and forms well-defined layers with molecules arranged along one direction. However, in some cases, a translational ordering within each layer may not exist, but the layers themselves constitute an additional degree of order. The molecules in the Sm mesophase are arranged in layers perpendicular to the director.^6−8^

Typically, polymeric materials with LC properties are obtained by embedding LCs in a polymeric matrix (polymer-dispersed LCs). The ordered fluid phases of LCs offer properties useful as precursors to high-performance polymer films, fibers, and injection molded items. Such systems are designed for numerous medical applications, such as artificial iris and blood sensors, as well as in the packaging industry in smart packaging as smart displays. For such applications, materials with LC properties must have low-temperature mesophase stability and a monomorphic stable mesophase capable of bistability.^9,10^ There are also thermotropic liquid crystal polymers (LCPs, formed by heating); these are thermoplastic polymers that can have a local molecular order (LC mesophase formation—mesogen). Semirigid polymer chains with local anisotropy have optical birefringence.^11^ The advantage of LCPs is that at RT they exist in a glass-like state, maintaining their molecular orientation. LCPs are of great interest because of their potential use as biomedical, photoelectric, and smart stimuli-responsive materials as well as thermally switchable light shutters.^12^ The transition between the different mesophases—from the crystalline, smectic, nematic-to-isotropic phase—takes place at specific temperatures that can be characterized by differential scanning calorimetry (DSC), while the nature and texture of the mesophases are examined by polarized optical microscopy (POM).^5^

The chiral nematic mesophase can also be obtained under mechanical stress or shear stress during the melt processing. Elevated pressure reversibly induces the formation of a LC state in mesogenic polymers. Under the influence of a slight shift of the plates (or layers), the confocal texture disappears and a planar texture appears in its place. The resulting flat texture, however, is unstable and slowly returns spontaneously to the confocal texture. Selforganized chiral nematic LCs can find practical applications in thermography (thermal mapping involving the visualization of color changes with the temperature) and electro-optics.^13^ Irreversible phase changes in virtually any polymers, even those without mesogenic structures deemed necessary to exhibit LC properties, can also be obtained under the influence of temperature and pressure.^9,14^ The method of inducing LC state involves applying high pressure to the polymer while heating it above or near its glass transition temperature (*T*_g_) but below its melting point (melting temperature, *T*_m_). This process provides a LC state that can be maintained for years under ambient conditions, even after the pressure is removed. (Bio)degradable polymers with such induced LC properties can be formed into films, film laminates, microparticles, coatings, membranes, and slabs and can also be extruded and molded.^9^

Polylactide (PLA) is a (bio)degradable linear aliphatic polyester synthesized from a biobased monomer mostly derived from sugarcane and corn starch. Thermoplastic PLA has high tensile strength and a high melting point, but low elongation at break because of its brittleness, which is the result of high crystallinity and *T*_g_ far above RT. The ratio of l- to d-enantiomers affects the properties of PLA such as the melting point and degree of crystallinity. Produced from renewable resources, PLA can be recognized as an advanced green material that has found applications in many areas, including the pharmaceutical, biomedical, and environmental sectors.^15^

Currently, a very important consideration when creating new polymer-based materials is not only their efficiency, but also whether they are environmentally friendly and green. Therefore, research is conducted on materials based on (bio)degradable polymers, such as PLA, polyhydroxyalkanoates (PHAs), poly(ε-caprolactone), or poly(butylene adipate-*co*-butylene terephthalate) with the addition of well-known and widely used LCs such as 4-cyano-4′-pentylbiphenyl (5CB) or copolymerized with polymers or copolymers containing the LC part in the polymer main- or side-chain such as the disclike polystyrene-*block*-poly(l-lactide) copolymer.^16−18^ In this case, however, the (bio)degradable polymers contain nonbiodegradable segments or additives. There are also cases describing the influence of tensile drawing or temperature and pressure on the morphology of biodegradable polymers. The ability to induce the mesophase by tensile drawing PLA at temperatures close to its *T*_g_ was described, which also improved the crystallization kinetics after annealing, as well as mesophase induced in nonmesogenic polymers by compression at controlled temperature.^19−26^ The characterization and quantification of two different PLA mesophases after pressure/temperature treatment of a nematic-like mesophase formed at temperatures below the *T*_g_ and a condis crystal-like mesophase formed at temperatures above the *T*_g_ were presented.^26^

This paper presents the LC properties of pressed predominantly poly(l-lactide) (PLLA) films with different textures obtained with changing processing parameters (under pressure at different temperatures and times). The materials studied were prepared from the initial PLLA rigid film, which was extruded and then thermoformed and, to confirm the repeatability of the LC properties, also obtained from the film by the solution casting method. It was observed that in the nonmesogenic thermoplastic polymer, LC properties can be induced by exposing the polymer to pressure at a temperature close to its *T*_g_. It has originally been shown that, by introducing talc, it is possible to obtain the colored planar Grandjean texture of a chiral nematic (N*) mesophase (visible under POM, atomic force microscope (AFM), and scanning electron microscope (SEM)).^9,27^ For the first time, the blue phase (BP) was also described in plain PLLA.

## Experimental Section

### Materials

Rigid PLLA films used as the initial PLLA with a d-lactide content of 5.8% (as estimated according to the previously described method based on the dependence of the melt temperature of PLLA films as a function of % d-isomer.^28^) and mass-average molar-mass *M*_w_ = 180,000 g mol^–1^ and molar-mass dispersity *Đ*_M_ = 2.0 (determined by gel permeation chromatography) were prepared by extrusion followed by thermoforming at the Institute for Engineering of Polymer Materials and Dyes (IMPiB Toruń, Poland) under the MARGEN project.^29^ During thermoforming, the polymer material underwent a deformation under the influence of stresses, and the state of strain was fixed during cooling, which gave it a specific thermal history (see Table 1). The granules used to obtain films by the solution casting method were a commercial PLA type 2002D, the product of Nature Works (Minnetonka, Minnesota, US). Talc powder (hydrous magnesium silicate), 10 μm, from Sigma-Aldrich (St. Louis, Missouri, US) were used as received.

**Table 1:** Thermal Properties of the Initial PLLA Rigid Film, Pressed PLLA Films Obtained at a Pressure of 5 tons for 1 min at 110 °C (Thread-Like Texture) and 50 °C (BPIII*), for 2 min at 100 °C (Colored Planar Texture), and Pressed PLLA/Talc Film with 0.5 wt % of Talc Obtained at a Pressure of 5 Tons for 1 min at 110 °C (Original DSC Traces in the Supporting Information, Figures S14–S18)^a^

sample	initial PLLA (nonmesogenic thermoplastic)	pressed PLLA (thread-like texture)	pressed PLLA/talc (colored planar texture)	pressed PLLA (colored planar texture)	pressed PLLA (BPIII*)
I-heating run at 20 °C·min^–1^					
enthalpic relaxation [°C]	67.2	63.4	62.4	68.7	65.8
*T*_cc_ [°C]	120.9	120.3		125.9	122.5
Δ*H*_cc_ [J g^–1^]	–21.79	–14.05		–8.85	–21.26
*T*_m_ [°C]	149.9	150.6	150.8	153.2/157.9	70.4/150.2
Δ*H*_m_ [J g^–1^]	22.22	24.62	36.44	9.15	1.48/21.37
cooling run at 10 °C·min^–1^ from melt					
*T*_g_ [°C]	54.3	54.8	54.1	54.5	54.6
Δ*c*_p_ [J g°C^–1^]	0.43	0.38	0.34	0.26	0.29
*T*_IN_ [°C]		147.0	142.8	143.4	144.3
Δ*H*_IN_ [J g^–1^]		0.20	0.12	0.23	0.18
*T*_c_ [°C]	101.5/129.7	138.9			115.7
Δ*H*_c_ [J g^–1^]	–0.36/–0.30	–0.02			–0.26
II-heating run at 10 °C·min^–1^ after cooling at 10 °C·min^–1^					
*T*_g_ [°C]	57.2^b^	59.8^b^	57.9^b^	59.2^b^	58.1^b^
Δ*c*_p_ [J g^–1^ °C]	0.54	0.55	0.57	0.47	0.46
*T*_m_^c^ [°C]	152.5	-	-	-	-
*T*_NI_^c^ [°C]	-	151.1	151.5	154.9	153.1
Δ*H*_m_^c^ [J g^–1^]	0.54	-	-	-	-
Δ*H*_NI_^c^ [J g^–1^]	-	0.19	0.60	0.25	0.28

a *T*_g_ is the glass transition temperature, Δ*c*_p_ is the the increment of heat capacity at the glass transition, *T*_m_ is the melting temperature, Δ*H*_m_ is the melting enthalpy, *T*_cc_ is the the maximum of the exothermic peak of the cold crystallization temperature, Δ*H*_cc_ is the cold crystallization enthalpy, *T*_NI_ is the nematic-to-isotropic transition temperatures, Δ*H*_NI_ is the nematic-to-isotropic transition enthalpy, *T*_c_ is the maximum of the crystallization peak, and Δ*H*_c_ is the crystallization enthalpy.

b Observed enthalpic relaxation.

c *T*_m_ and Δ*H*_m_ for nonmesogenic thermoplastic polymer, *T*_NI_ and Δ*H*_NI_ for LCs.

### Pressed Film Preparation

Pressed PLLA films, with a mean thickness of 0.31 ± 0.02 mm, were prepared from cut strips of rigid films (initial PLLA) with mean dimensions of 20 × 15 ± 2 mm on a hydraulic press with a force from the pressure of the press jaws up to 5 tons at a temperature of the press heating plate from RT to 140 °C for 1 to 5 min. The temperatures for the preparation of the PLLA films with LC properties were experimentally selected from the lowest, where the LC state occurred to the temperature, where the LC state was not obtained. Other parameters were chosen similarly.

PLLA/talc samples with 0.1 and 0.5 wt % of fine powder were pressed into films with a force of 5 tons at 110 °C for 1 min. Talc was spread over the entire surface of the initial PLLA rigid film. Additionally, PLLA films were obtained by the solution casting method. PLLA was dissolved in chloroform to obtain a concentrated solution (10 wt %), which was then used to cast the films onto Teflon disks. Thus-obtained films were used to obtain pressed PLLA films to confirm the repeatability of obtaining polymers with LC properties.

### Characterization

The textures of the mesophase were observed with a polarized optical microscope Zeiss (Opton-Axioplan) equipped with a Nikon Coolpix 4500 color digital camera and a Mettler FP82 hot plate with a Mettler FP80 temperature controller. The sample was placed on a microscope slide with a cover slip, and then the slide was heated and cooled while observing the phase changes. SEM studies were performed using Quanta 250 FEG (FEI Company, USA) high-resolution environmental SEM operated at 5 kV acceleration voltages. The samples were observed without coating under a low vacuum (80 Pa) using a secondary electron detector (large field detector). Atomic force microscopy measurements were performed using a Dimension ICON AFM microscope equipped with a NanoScope V controller (BRUKER Corporation, Santa Barbara, CA, USA) operating in the soft tapping mode in an air atmosphere with a standard 125 μm long and 10–15 μm high tip, with a flexural stiffness of 42 N·m^–1^ of single-crystal-doped silicon cantilevers (Model RTESP-300, BRUKER, Camarillo, CA, USA). Images were obtained with a piezoelectric scanner with a nominal size of 85 × 85 μm. The micrographs were recorded using NanoScope Analysis 1.9 Software (BRUKER Corporation, Santa Barbara, CA, USA). The most representative images for each film were selected from three measurements taken on several films. The thermal characteristic of the samples was obtained using the DSC Q2000 apparatus (TA Instruments, Newcastle, DE, US). The instrument was calibrated with high-purity indium. The first heating runs concerned initial samples in which the thermal history is suppressed. DSC studies were carried out at a temperature from −90 to 200 °C with a rate of 20 (I-heating run) and 10 °C·min^–1^ (cooling and II-heating run). All of the experiments were performed under a nitrogen atmosphere with a nitrogen flow run of 50 mL·min^–1^, using aluminum standard sample pans. The *T*_m_ was taken as the peak temperature maximum of that melting endotherm, and the *T*_g_ was taken as the midpoint of the heat capacity change of the sample. X-ray diffraction and residual stress analysis were performed using a D8 advance diffractometer (Bruker, Karlsruhe, Germany) with a Cu-Kα cathode (λ = 1.54 Å). The scan rate was 1.2°·min^–1^ with a scanning step of 0.02° in the range of 5° to 60° 2θ using Bragg–Brentano geometry, while residual stress analysis was performed using grazing incidence geometry with an incidence angle of 1°. All measurements were performed in triplicate. Identification of fitting phases was performed using the DIFFRAC.EVA program with the ICDD PDF#2 database, while the crystalline size, lattice strain, and lattice parameters of *P*2_1_2_1_2_1_ orthorhombic PLLA crystallites were calculated using Rietveld refinement in the TOPAS 6 program, based on Williamson–Hall theory.^30−32^ The pseudo-Voigt function was used in the description of diffraction line profiles at the Rietveld refinement. The weighted-pattern factor and goodness-of-fit parameters were used as numerical quality criteria for the fit of calculated experimental diffraction data. The shift of peaks due to residual stress was calculated according to eq 1 using the TOPAS 6 program. The following material parameters were adopted for the analysis of residual stress: Young modulus *E* = 3500 MPa and Poisson ratio ν = 0.33. Stress-free PLLA was calculated at the 0.25 MPa level.
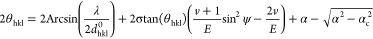
1where 2θ_hkl_ is the peak position, λ is the wavelength of the source, *d*^0^_hkl_ is the interplanar spacing, σ is the stress, *ν* is the Poisson ratio, *E* is the Young’s modulus, ψ is the crystallite orientation, α is the incidence angle, and α_c_ is the critical angle.

## Results and Discussion

### LC Behavior

The nematic mesophase was obtained under the influence of temperature and mechanical stress caused by pressing. First, a PLLA rigid film was heated from RT to a temperature below its *T*_m_ = 149.9 °C (from 20 to 110 °C, see Table 1) under the pressure of the press jaws to 5 tons for about 1 to 5 min, and then the PLLA was cooled to a temperature below the *T*_g_ = 54.3 °C (i.e., RT), releasing the pressure of the jaws. Additionally, the chiral nematic mesophase was obtained by appropriate control of the temperature, heating time, and pressure force, as well as by adding a fine powder, for example, talc (also peptides or cyclodextrins; publications in preparation^33,34^). When the sample was cooled to RT, the polymer retained the structure of the nematic mesophase.^7,9^ Slow plastic deformation, which is the pressing of the polymer in the temperature range between *T*_g_ and *T*_m_, facilitates ordering, as the straightening (orientation) of the chains takes place during the deformation. Pressure and temperature increase chain mobility, which improves order in macromolecules and causes a disorder/order transition.

**Figure 1:**
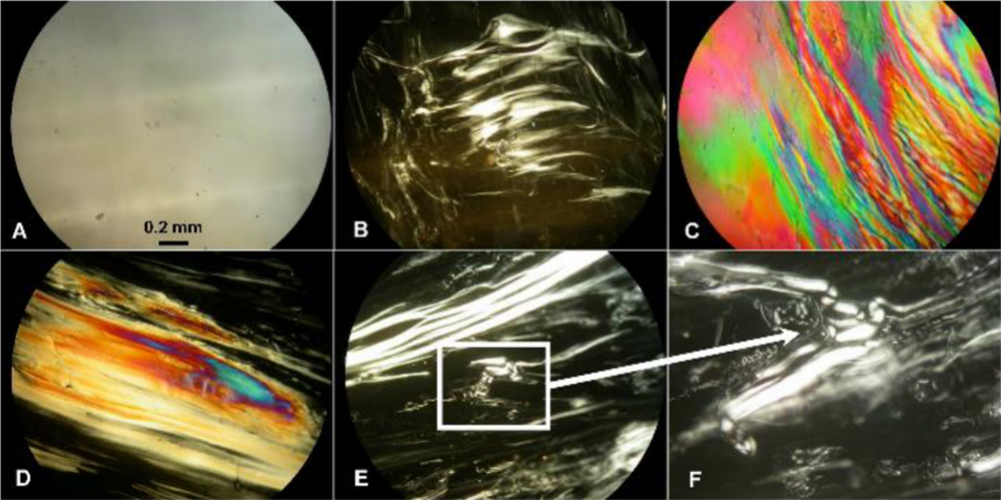
Representative photomicrographs of optical textures of the nonmesogenic thermoplastic initial PLLA film (A), nematic mesophase of the pressed PLLA film (B), and colored planar texture of the chiral nematic mesophase of the pressed PLLA/talc film with 0.5 wt % of talc (C), as well as colored planar texture (D) and schlieren texture (E and F—enlarged image showing topological defects) of the nematic mesophase of the pressed PLLA/talc film with 0.1 wt % of talc. Pressed films were obtained at a pressure of 5 tons for 1 min at 110 °C (crossed polarizers, 25 °C, 100×).

Phase behavior of tested films obtained at the same pressure, time, and temperature was examined by POM observation of the optical texture and is presented in Figure 1.

The nematic mesophase, when viewed under a POM between crossed polarizers, created distinctive dark thread-like structures (Figures 1B and S1 in the Supporting Information) that are topological defects. Defects in LC systems are important for the identification of mesophase types.^35^ In the chiral nematic mesophase, the molecules twist perpendicular to the director axis (axis of rotation), with the molecular axis parallel to the director. The twist angle between adjacent molecules results from the asymmetric packing leading to a longer-range chiral order. The distance along the helical pitch for a full rotation of the mesogens is a strong function of the temperature. Generally, the helical pitch of cholesteric LCs is of the order of several hundred nanometers (the wavelength of visible light) and thus exhibits interference colors.^6−8^ In POM, the isotropic phase is dark under crossed polarizers, while the birefringent nematic mesophase exhibits interference colors. The bright colors are due to the difference in rotatory power resulting from domains with different cholesteric pitches.^5^ The schlieren texture of an N phase is observed for a flat sample between crossed polarizers, showing a network of black brushes connecting centers of point and line defects (Figures 1E,F and S4 in the Supporting Information).^36,37^ This texture is observed in a planar cell, where the director aligns parallel to the surface and is organized around point disclinations, surface disclination lines, and inversion walls.^38^

In the case of the N* mesophase under POM, interference colors of the planar texture, also called Grandjean texture of the N* mesophase (the most stable and that with the lowest energy state), were visible (Figures 1C and S9 in the Supporting Information).^39−42^ Grandjean texture is observed when the helical axis in the layer with perfect planar texture is perpendicular to the boundary surface. The planar texture is illuminated by white light and shimmers with colors, which changes according to the viewing angle. The planar Grandjean texture of the N* mesophase is obtained similar to the planar texture in ordinary nematic, that is, by imposing boundary conditions such that the molecules in contact with the bonding surfaces are aligned parallel to these surfaces. The resulting texture is durable and can last for a long time, many months, or even years.^43−47^

The N* mesophase was also obtained by introducing a small amount (0.5 wt %) of talc into the polymer matrix, suggesting that a fine powder of talc acted as a nucleation agent, which increases order and facilitates the twist of the molecules perpendicular to the axis of rotation (Figures 1C and S2 in the Supporting Information). Talc as a potential nucleating agent is a widely used additive to polymers. It is chemically inert, soft, and a water repellent as well as having an affinity for organic substances. In the case of PLA, the addition of talc accelerates ordering and increases the nucleation density (entropic effect).^48,49^ It was also observed that the amount of powder added is important for the type of texture obtained. The addition of 0.1 wt % resulted in the heterogeneity of the material and the formation of areas with different textures. A smaller amount of talc is more difficult to distribute across the surface, hence the heterogeneity resulting from more dispersed nucleation. Coexistence of dark and bright domains with a colored planar texture (Figures 1D and S3 in the Supporting Information) and a schlieren texture of the N mesophase (Figures 1E,F and S4 in the Supporting Information) was observed.^50^

**Figure 2:**
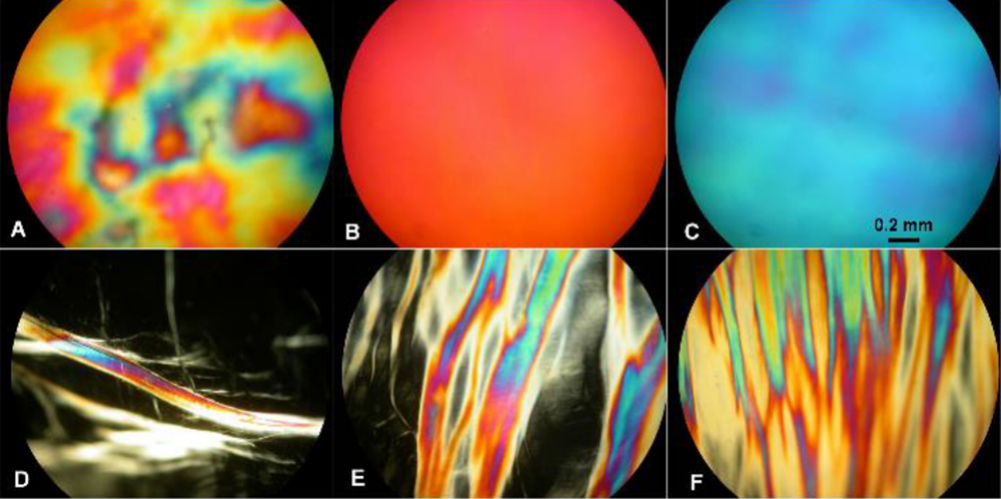
Representative photomicrographs of optical textures of the pressed PLLA film obtained in different processing parameters: at a pressure of 5 tons for 1 min at 20 °C (RT, color fog texture, A), 40 and 50 °C (BPIII*, B and C, respectively), for 2 and 3 min at 110 °C (nematic mesophase with a heterogeneous surface, D and E, respectively), and for 5 min at 110 °C (colored planar texture of the chiral nematic mesophase, F) (crossed polarizers, 25 °C, 100×).

By selecting the appropriate processing parameters (temperature, heating time, and pressure force), it is possible to obtain not only a nematic mesophase, but also the selected texture. Several types of nematic textures have been observed, such as thread-like texture, schlieren texture of the N mesophase, oily streak texture, planar Grandjean texture of the N* mesophase, fog texture, and amorphous blue phase (BPIII*) (see Figures 1, 2, and S1–S11 in the Supporting Information).

As it turned out, LCs are also formed below the *T*_g_ of the polymer (at RT), because for, an adiabatic process in which there is no heat transfer with the environment, the temperature of the pressed material increases during pressing. Therefore, already at RT, under the pressure of 5 tons for 1 min, the fog texture of the amorphous pressed PLLA film can be obtained (Figure 2A, see also Table 1 with thermal properties). Other types of textures appeared as the processing temperature increased. A BP of the amorphous pressed PLLA film was obtained between 30 and 60 °C (Figures 2B,C and S5 in the Supporting Information). The BP appears in nematic LCs with a strong helical twisting forcer that may exhibit different crystalline phases (crystalline BPI* and BPII*, as well as amorphous BPIII*). The temperature range over which the BP occurs is usually very narrow and therefore difficult to observe as the BPs are highly fluid, self-assembled three-dimensional cubic defect structures that exist in narrow temperature ranges, between the isotropic (liquid) and cholesteric LC phases, in highly chiral LCs.^27^ However, BPs are not always blue. They can also reflect the light of other colors, including near infrared.^51^ In the case of the pressed PLLA film, this phase was observed by lowering the processing temperature below 70 °C, also with lower pressure (1 ton) and longer time (2 min). At 70 °C, the fog texture was again obtained, while at 80 °C a heterogeneous film was obtained with areas of different textures (thread-like and colored planar texture of N* mesophase, see Figures S6 and S7 in the Supporting Information). This heterogeneity occurred irrespective of the pressure (even under the pressure of press jaws but for 2 min, see Figure S8 in the Supporting Information). At a processing temperature close to *T*_m_ = 148.9 °C (see Table 1), only dark and bright domains with a thread-like texture appeared (Figures 1B and S1 in the Supporting Information). Longer pressing times were required at these temperatures to obtain colored planar texture of the N* mesophase; 2 min for 80 and 100 °C as well as 5 min for 110 °C (Figures 2F and S8 in the Supporting Information). At intermediate processing times (1 min for 80 °C and 2–4 min for 110 °C) a heterogeneous film was obtained (Figures 2D,E, S7, S10, and S11 in the Supporting Information). At a processing temperature of 140 °C, the pressed PLLA films no longer exhibit LC properties. The possibility of obtaining different textures by selecting different processing parameters for the pressed PLLA film obtained from the initial PLLA rigid film, which was extruded and then thermoformed, was confirmed for the pressed PLA films obtained by other methods—solution casting. The experiments confirm the repeatability of obtaining polymers with LC properties (Figures S12 and S13 in the Supporting Information).

To confirm the nematic mesophase, the thermal behavior of the obtained materials was investigated. The thermal properties of the initial PLLA rigid film, pressed PLLA film obtained with different processing parameters, and pressed PLLA/talc film with 0.5 wt % of talc were evaluated by the DSC method (Table 1).

In the first heating run in which thermal history is suppressed for the initial PLLA rigid film (20 °C·min^–1^, Table 1), the glass transition and possible melting transition overlapped with the strong structural relaxation, which indicates that the amorphous polymer chains were “frozen” into high-energy conformation during fast cooling as part of the rigid film processing (extrusion and thermoforming processes).^52^ DSC analysis shows that the initial PLLA rigid film and pressed PLLA film obtained at a pressure of 5 tons for 1 min at 50 °C (BPIII*) and for 2 min at 100 °C (with a colored planar texture of N* mesophase) are amorphous, and pressed PLLA films obtained at 110 °C (with thread-like texture) are partially ordered as well as pressed PLLA/talc film with 0.5 wt % of talc partially crystalline (see cold crystallization enthalpy (Δ*H*_cc_) and melting enthalpy (Δ*H*_m_) in Table 1). A complex melting pattern with two endotherms located at 70.4 and 150.2 °C, respectively, was observed for the pressed PLLA film with the BP. The endo-exo transition was observed at 70.4 °C despite the amorphous nature of the polymer. A multiple melting peak was also observed for the pressed PLLA/talc film with 0.5 wt % of talc. The complex melting pattern is a common phenomenon with polymers. This may indicate the presence of several distinct crystal populations, but it is more likely to be due to different crystal morphology and microstructure changes (lamellar thickness or crystal perfection) prior to the onset of total phase change during melting because of the addition of talc.^53^ For all samples, an endothermic phenomenon, strong structural relaxation effect, caused by enthalpic relaxation of the amorphous glassy state from unstable chain conformations toward a more stable state overlapped by PLLA glass transition relaxation at about 66 ± 2.6 °C was observed. Enthalpic relaxation was also observed in the second heating run for all samples. During the first heating run, a broad exothermic peak was observed at about 122 ± 2.5 °C with mean enthalpy Δ*H*_cc_ = −22 ± 0.4 J g^–1^ for both the amorphous initial PLLA rigid film and pressed PLLA with BP, while with Δ*H*_cc_ = −8.84 J g^–1^ for pressed PLLA with colored planar texture and Δ*H*_cc_ = −14.05 J g^–1^ for partially ordered pressed PLLA with thread-like texture. A melting endotherm was also observed at about 151 ± 1.3 °C for all samples with mean enthalpy Δ*H*_m_*=* 22 ± 0.6 J g^–1^ for both the amorphous initial PLLA rigid film and pressed PLLA with the BP, while with Δ*H*_m_*=* 9.15 J g^–1^ for pressed PLLA with colored planar texture, Δ*H*_m_*=* 24.62 J g^–1^ for pressed PLLA with thread-like texture, and Δ*H*_m_*=* 36.44 J g^–1^ for the pressed PLLA/talc film with 0.5 wt % of talc (no cold crystallization). The glass transition temperature of all PLLA films at about 58 ± 1 °C was taken from the second heating run with the rate of 10 °C·min^–1^ (Table 1).

**Figure 3:**
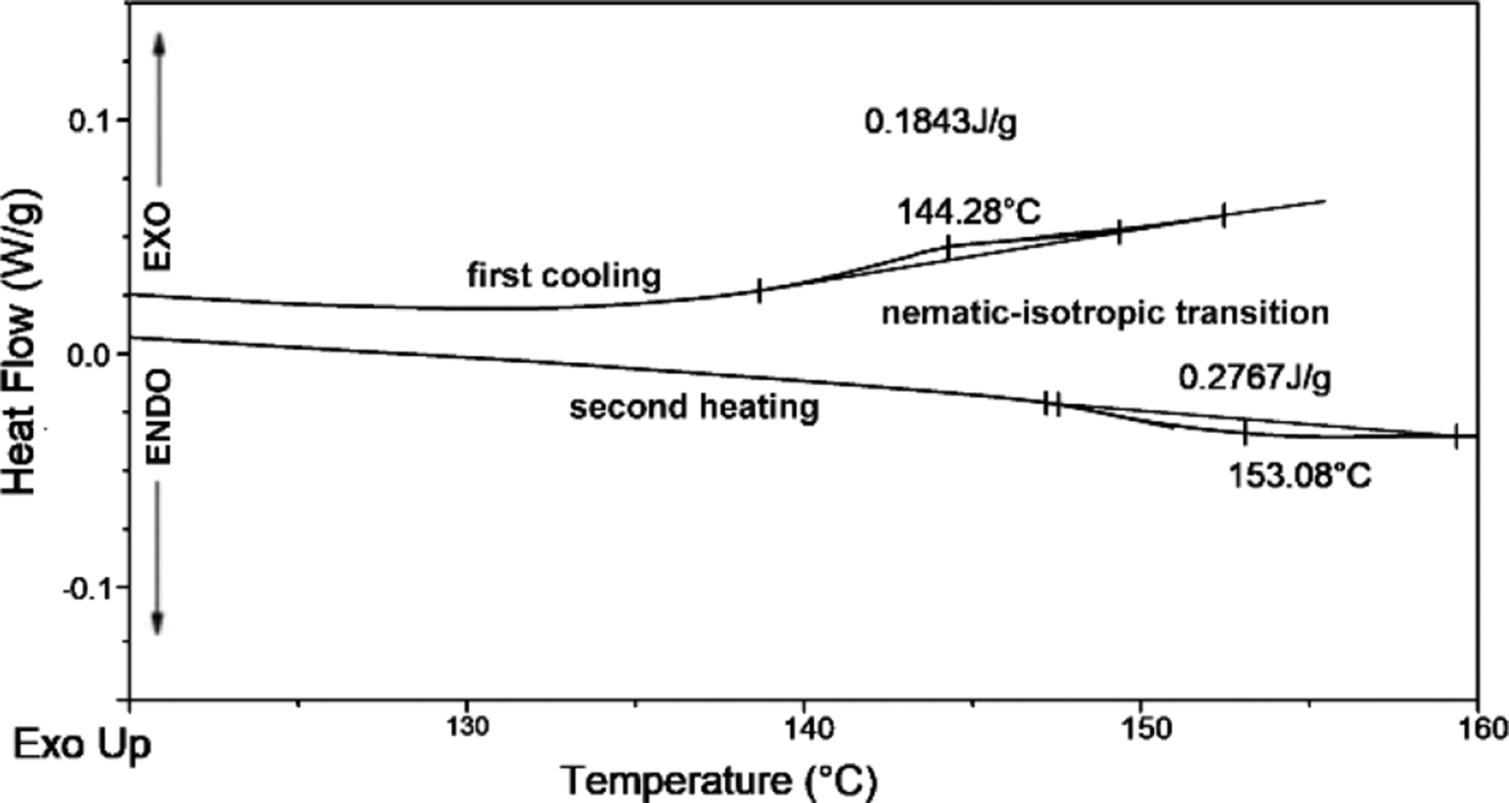
Representative DSC traces of the amorphous pressed PLLA film with the BP obtained at 10 °C·min^–1^ in the cooling run and second heating run.

Representative DSC traces of the amorphous pressed PLLA film obtained at a pressure of 5 tons for 1 min at 50 °C (BPIII*) during cooling and second heating runs with a rate of 10 °C·min^–1^ are presented in Figure 3. The sample was first heated from −90 to 200 °C at a rate of 20 °C·min^–1^ to eliminate the effect of thermal history.

The drawback of materials with the BP is the limited thermal stability between isotropic and N* thermotropic phases (0.5–2 °C), which limits their practical application. A wider temperature range would potentially open up new possibilities for photonic applications.^27^ Upon DSC analysis with the rate of 10 °C·min^–1^, the pressed PLLA films showed only *T*_g_ and mesophase to isotropic state transition (nematic-to-isotropic transition, NI; Table 1, Figure 3). The pressed PLLA film with the BP exhibited a nematic-to-isotropic transition temperature at 153.1 °C (Δ*H*_NI_ = 0.28 J g^–1^) and isotropic to the nematic temperature at 144.3 °C (Δ*H*_IN_ = 0.18 J g^–1^) during heating and cooling runs, respectively. For the BP, Δ*T* ≈ 9 °C, which is a certain extension of the temperature range. The pressed PLLA/talc film with 0.5 wt % of talc with the colored planar texture of the N* mesophase exhibited *T*_IN_ at 142.8 °C (Δ*H*_IN_ = 0.12 J g^–1^) and *T*_NI_ at 151.5 °C (Δ*H*_NI_ = 0.60 J g^–1^) during cooling and heating runs, respectively. In the case of the cooling run, for some PLLA films, there was a crystallization temperature (*T*_c_); however, this effect was small with mean crystallization enthalpy Δ*H*_c_ = 0.1 ± 0.2 J g^–1^ and did not repeat in the following cooling runs. The initial PLLA rigid film has crystallization temperature during cooling, which, however, was located at a lower value and with higher Δ*H*_c_ (*T*_c_ = 101.5 and 129.7 °C; Δ*H*_c_ = 0.36 and 0.30 J g^–1^).

**Figure 4:**
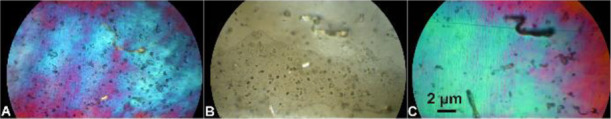
Reversible nematic-to-isotropic transition of the pressed PLLA film with the BP. Photomicrographs of the optical texture of the chiral nematic enantiotropic mesophase during heating and cooling at 115 °C (nematic mesophase, A), 150 °C (isotropic phase, B), and 144 °C (nematic mesophase after cooling, C) (crossed polarizers, 160×).

DSC investigations revealed that the pressed PLLA films exhibited LC properties. Generally, polymers with thermotropic LC properties show phase transitions in the bulk state when heated. The LCs pass from the crystalline state (positional and orientational order) through the mesophase (orientational order only) to an isotropic liquid state (disordered). Usually, mesophases are fairly narrow and can only appear on cooling or heating (monotropic mesophases). If mesophases occur during heating and cooling, they are referred to as enantiotropic. Heating the sample under the microscope and observing the texture changes during the phase transition allow for the identification of the transition temperature and additionally indicate the type of mesophase, as the texture and structure of defects are characteristic of a given mesophase.^8^ LC polymers generally do not exhibit polymesomorphism because of their high molar mass. Additionally, in polymers, a nematic-to-isotropic transition is often not observed because thermal degradation of the polymer usually precedes this transition. Identifying the correct LC mesophase and texture is also sometimes difficult for polymers where both amorphous and crystalline domains coexist.^54^ The nematic phase has been confirmed by all three techniques together (POM, DSC, and X-ray); however, the most important evidence of mesogenic behavior is the observation of phase transitions as a function of temperature under POM. Consequently, the mesophase behavior of the pressed PLLA film was also confirmed by observation of the optical textures on POM equipped with the hot stage (Figures 4 and S19–S21 in the Supporting Information).

Figure 4A illustrates the nematic enantiotropic mesophase of the pressed PLLA film with the BP under crossed polarizers at 115 °C. By increasing the temperature to 150 °C, the nematic enantiotropic mesophase lost its birefringence and was transformed into an isotropic phase and the texture turned dark (Figure 4B). When the polymer was heated to the isotropic state, there is no long-range positional or orientational order, while mesophases are achieved when the sample is cooled.^8^ After cooling the isotopic phase to 144 °C, the nematic mesophase with BP texture forms again (Figure 4C). DSC studies reveal the presence of LC mesophases by detecting changes in enthalpy associated with the phase transitions and the amount of released or absorbed energy. The lower energy transitions are usually associated with the nematic mesophase, while the higher energy transitions are associated with the smectic and crystalline phases.^37^ The POM observation of the optical texture of mesophase (BPIII*), first heated and then cooled, as well as the reversible nematic-to-isotropic phase transition and low enthalpy of this transition, (Δ*H*_NI_ = 0.18 J g^–1^), found in DSC experiments suggests that pressed films form a stable N* enantiotropic mesophase (Figures 3 and 4).

**Figure 5:**
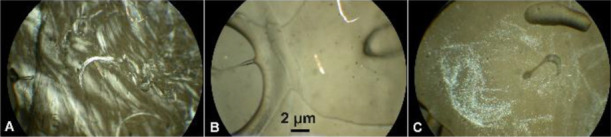
Irreversible nematic-to-isotropic transition of the pressed PLLA film with thread-like texture. Photomicrographs of the optical texture of the nematic monotropic mesophase during heating and cooling at 145 °C (nematic mesophase, A), 150 °C (isotropic phase, B), and 90 °C (crystallization during cooling, C) (crossed polarizers, 160×).

In the case of pressed PLLA films with a thread-like texture, an irreversible monotropic nematic mesophase was obtained (Figure 5).

**Figure 6:**
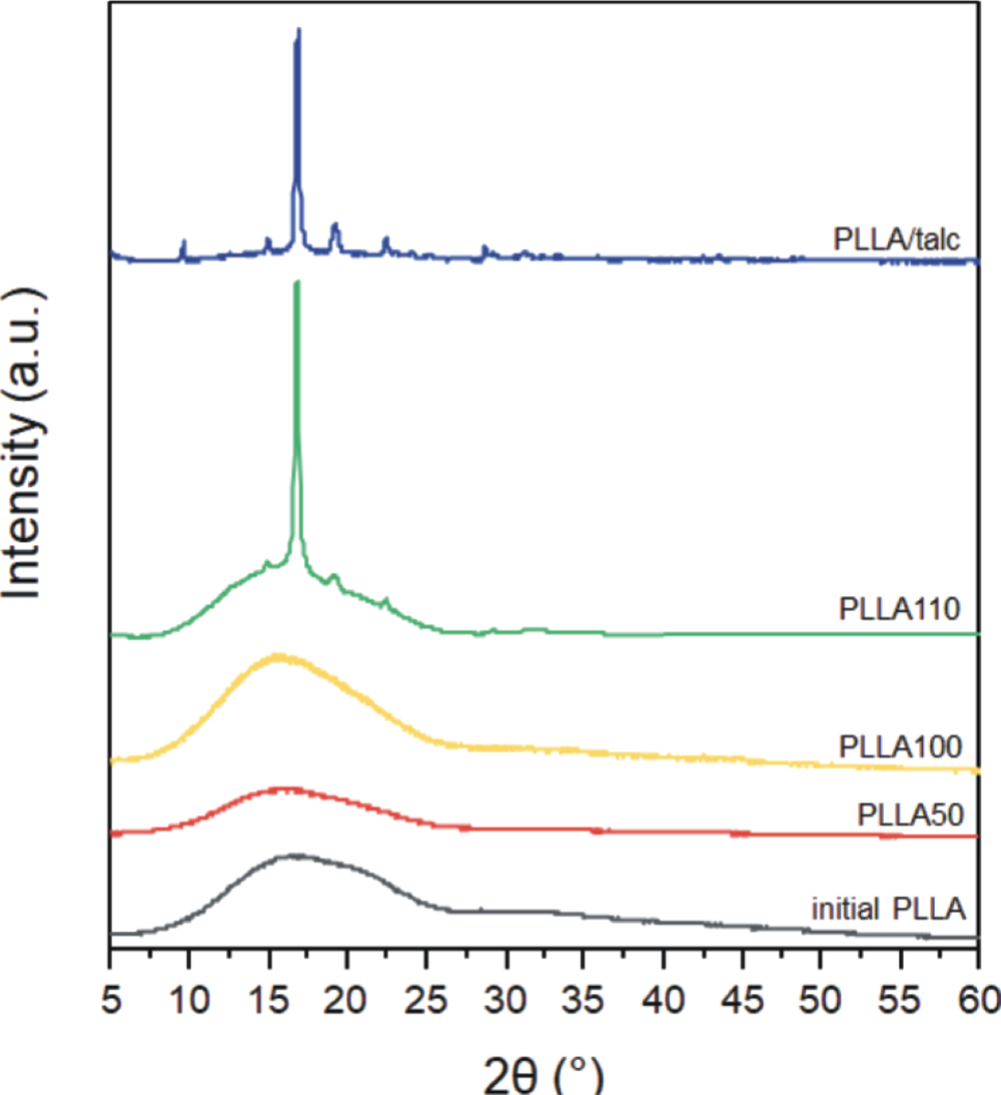
Representative X-ray diffractograms of the initial PLLA rigid film, pressed PLLA films obtained at a pressure of 5 tons for 1 min at 110 °C (thread-like texture, PLLA110) and 50 °C (BPIII*, PLLA50), for 2 min at 100 °C (colored planar texture, PLLA100), and pressed PLLA/talc film with 0.5 wt % of talc obtained at a pressure of 5 tons for 1 min at 110 °C. All scans were normalized to the maximal intensity of the initial PLLA.

In addition, an X-ray diffraction analysis of the initial PLLA rigid film, pressed PLLA films obtained with different processing parameters, and pressed PLLA/talc film with 0.5 wt % of talc was performed to confirm the presence of the nematic mesophase (Figure 6).

A broad scattering pattern can be seen for the initial PLLA, pressed PLLA with colored planar texture, and pressed PLLA with BPIII* attributed to the reflection of the amorphous phase in the 2θ region 8°–26°.^55^ On the contrary, the pressed PLLA/talc film with 0.5 wt % of talc obtained at 110 °C with colored planar texture shows very sharp diffraction peaks typical of the PLLA crystalline profile, indicating a reduced amorphous fraction, respectively 2θ = 14.9°, 16.8°, 19.2°, and 22.5° corresponding to the lattice planes (010), (110)/(200), (203), and (015) of the α-form of the PLLA crystallites.^56^ It is well documented in the literature that the α-form crystallizes spontaneously, while the growth of the α′-form is strain-activated, as an effect of polymer chain stretching.^57,58^ Because of relatively low stress in examined samples and the manufacturing process of PLLA samples, it is unlikely to observe the presence of the α′-form, which was also confirmed by DSC results (see Table 1). Moreover, some peaks typical of monoclinic talc with space group *C*2/*c* were detected around the 2θ of 10° and 28°.

The diffractogram of the pressed PLLA with thread-like texture obtained at 110 °C was found to differ significantly from that of initial PLLA and slightly from that of the pressed PLLA/talc film. The diffractogram of the pressed PLLA film obtained at 110 °C with a thread-like texture shows one dominant peak at 16.8° superimposed on the amorphous halo, suggesting that the signals originate predominantly from the mesomorphic phase characterized by a degree of organization ranging in a wide region between the crystalline and liquid states.^58^

The analysis of the X-ray pattern of the pressed PLLA with thread-like texture at 25 °C (Figure 6, PLLA110) shows the presence of the reflections at wide angles confirming a crystalline fraction and an amorphous halo in the 2θ region 8°–26° on the flat film diffractogram, which arises from the interchain spacing and is responsible probably for N* mesophase existence.^59^ It is known that a nematic mesophase only shows a diffuse halo in a high-angle region.^60,61^ The X-ray analysis confirmed the results of DSC and POM investigations and indicated that the obtained materials exhibited both crystalline and nematic behavior. However, detailed information about the molecular organization within the various phases at different temperatures can be obtained by X-ray diffraction measurements as a function of the temperature. Such experiments are necessary to define in detail the type of the nematic mesophase.^62^ On the other hand, the analysis of the pressed PLLA/talc (Figure 6, PLLA/talc) shows the presence of only reflections at wide angles, which indicates the predominant presence of the crystalline fraction.

The lattice parameter as a function of temperature and composition is important information for modeling the evolution of the material microstructure. This parameter relates to the physical dimension of the unit cells in the crystal lattice. Orthorhombic lattices, such as *P*2_1_2_1_2_1_ typically reported for PLLA, are characterized by three lattice constants, referred to as *a*, *b*, and *c*.^63^Table 2 shows the crystallographic data of the pressed PLLA films obtained with different processing parameters and pressed PLLA/talc film with 0.5 wt % of talc.

**Table 2:** Lattice Parameters, Crystallite Size, Lattice Strain, and Residual Stress Determined for the Pressed PLLA Films Obtained at a Pressure of 5 tons for 1 min at 110 °C (Thread-Like Texture, PLLA110) and Pressed PLLA/Talc Film with 0.5 wt % of Talc Obtained at a Pressure of 5 tons for 1 min at 110 °C

	lattice parameters [Å]						
sample	a	b	c	lattice volume [Å^3^]	crystallite size [nm]	lattice strain [%]	residual stress [MPa]
reference PLLA^a^	10.84	6.19	28.95	1942.5			
PLLA110	10.72	6.21	29.16	1940.8	41 ± 8	0.05 ± 0.01	4.8 ± 1.0
pressed PLLA/talc	10.82	6.07	28.85	1894.8	75 ± 5	–1.16 ± 0.05	–3.95 ± 0.8

a From International Center for Diffraction Data, ICDD #00-064-1623.

The physical meaning of the negative numbers of lattice strain and residual stress indicates their compressive nature, while the positive value indicates the tensile nature of the stress in the crystallites, as well as in the meaning of lattice strain. Because of the amorphous nature of PLLA with colored planar texture and pressed PLLA with BPIII*, resulting in the absence of the PLLA crystal, lattice parameters, crystallite size, and residual stress for those samples were not calculated. Lattice parameters of the pressed PLLA with thread-like texture in comparison with the model data show a slight reduction of the parameter a (1.1%), but the parameters b and c enlarge, 0.3 and 0.7% respectively. Lattice volume hardly changes, which with near zero lattice strain suggests the contribution of only linear stress. The value of the residual stress is high and shows a tensile nature. Compared with the model data for the reference PLLA and values of the pressed PLLA with thread-like texture, PLLA/talc shows changes of the parameter a (0.2% reduction and 0.9% enlargement, respectively) and the reduction of parameter c (0.3 and 1.1%, respectively) of the orthorhombic *P*2_1_2_1_2_1_ unit cell. It should be noted that the highest change was calculated for parameter b (reduction of 1.9%) compared to reference PLLA, with a simultaneously strong change of the lattice volume, which suggests the contribution of compressive stress.

The relative crystallinity of the PLLA matrix, calculated by the peak decomposition method, for pressed PLLA with thread-like texture is 1.6% and for PLLA/talc 36.6%. The use of talc as nucleating agents for PLLA to provide a strong increase in the polymer crystallinity has been previously described.^53^ The obtained results suggest that the introduction of the talc particles into the PLLA matrix caused shear stress during the crystallization process and induced specific nematic textures.

### Surface Characteristics

The LC arrangement is usually described by a positional arrangement in an ordered lattice and orientational order (local molecular order is mainly directed in one direction). The degree of order of the mesophase is on a molecular scale and often extends over micrometer-sized domains. Rod-like mesogens can exhibit a nematic mesophase with rods parallel to each other;^8^ therefore, the surface of the obtained films was examined using SEM and AFM techniques.

**Figure 7:**
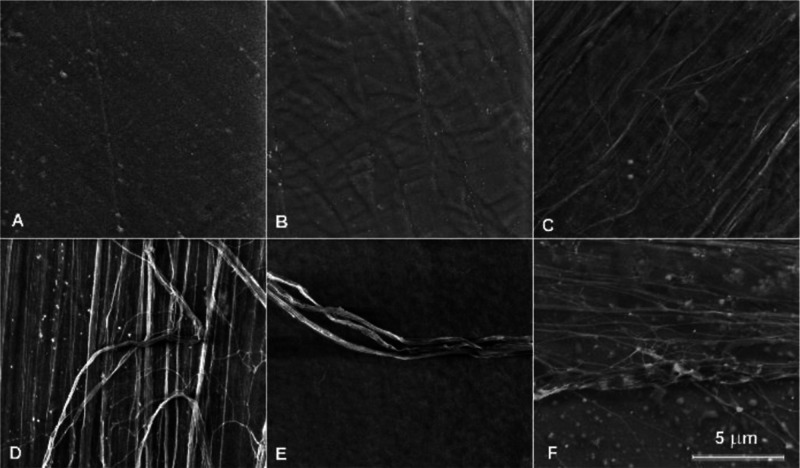
Representative SEM images of the initial PLLA rigid film (A), pressed PLLA films obtained at a pressure of 5 tons for 1 min at 50 °C (BPIII*, B) and 110 °C (thread-like texture, C) of 1 tons for 2 min at 100 °C (colored planar texture, D and E) and pressed PLLA/talc film with 0.5 wt % of talc (colored planar texture, F).

SEM examination of the surface of the pressed films revealed a characteristic local molecular order in the nematic mesophase with different textures (Figure 7).

The obtained SEM image of the BP shows many small multiplatelet domains (Figure 7B). The BP can be described as a combination of double-twisted cylinders and disclinations. Those cylinders are arranged in a cubic crystal structure with a cubic center or a simple cubic symmetry or exhibit a disordered structure. The micrograph shows the areas surrounded by concentric lines. They correspond to the dislocation lines resulting from the deformation caused by the slanting phase surface.^64^

SEM studies of the surface of the pressed film obtained under the pressure of 5 tons for 1 min at 110 °C revealed thread-like structures characteristic of the local molecular order in the N mesophase, which is arranged parallel to their long axes (Figure 7C). SEM images also revealed fibril agglomeration. They appear on the surface of the film, twisting, splitting, bending, and aggregating or intertwining.^65^ In the N* mesophase, the mesogens additionally twist perpendicular to the director, with the molecular axis parallel to the director. In this case, the agglomerations of fibrils are more twisted (Figure 7D–F). Figure 7F shows the characteristic surface disclination lines in the planar Grandjean texture of N* mesophase.^32^ Interestingly, the SEM image (Figure 7F) reveals spheres with a mean diameter of 213 ± 93 nm. Those spheres are likely the result of the phase separation between the LC rigid mesophase and the relatively soft part of the polymer.^66^

**Figure 8:**
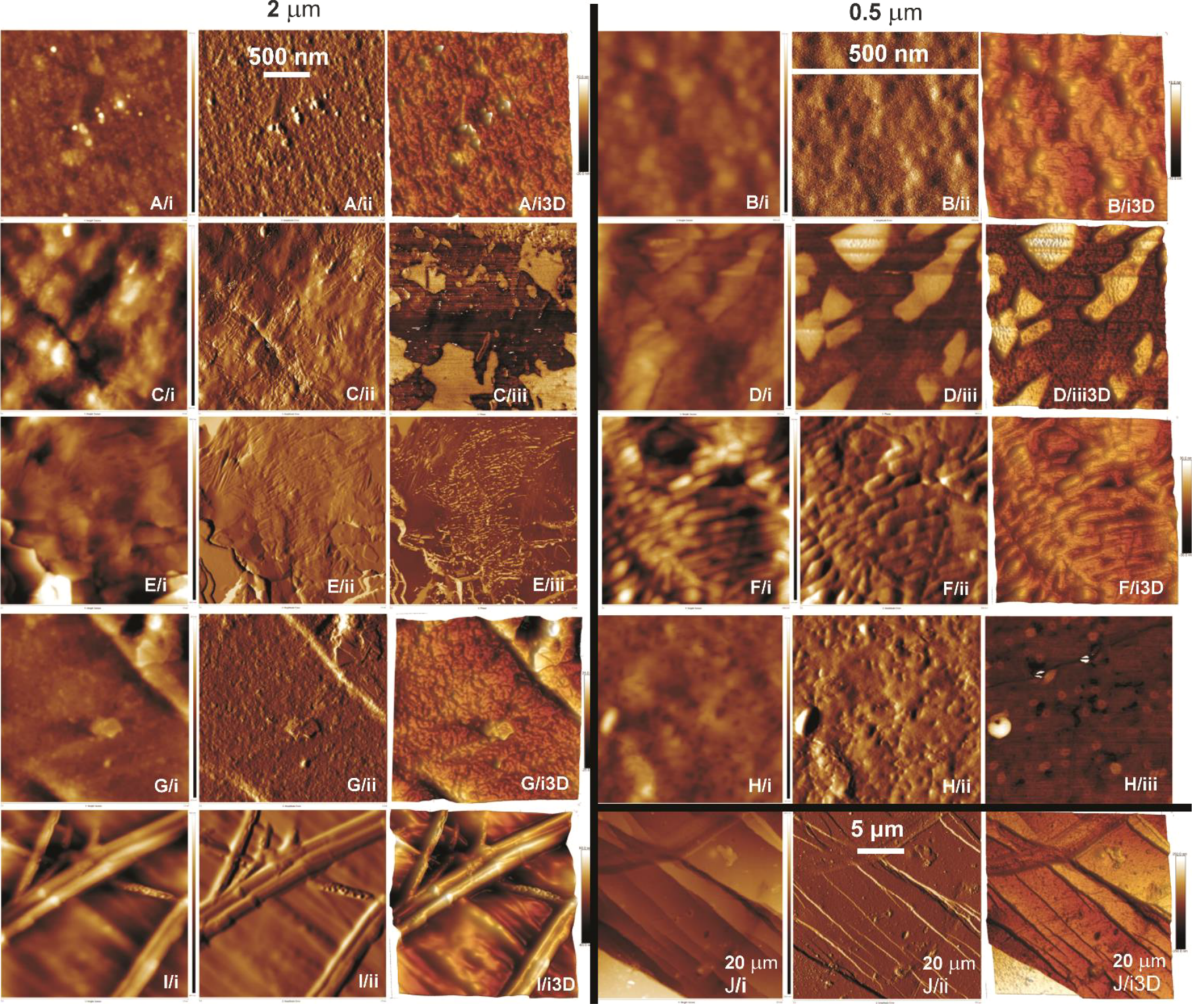
Representative AFM with a scan size of 0.5 × 0.5, 2 × 2, and 20 × 20 μm height sensor (i), amplitude error (ii), phase (iii), and 3D (i3D and iii3D)) images of the initial PLLA rigid film (A and B), pressed PLLA films obtained at a pressure of 5 tons for 1 min at 110 °C (thread-like texture, C), for 2 min at 100 °C (colored planar texture, D), for 5 min at 110 °C (colored planar texture, I), pressed PLLA/talc film with 0.5 wt % of talc (colored planar texture, E and F), and pressed PLLA films obtained at a pressure of 5 tons for 1 min at 50 °C (BPIII*, G, H, and J).

There are several different types of images that AFM analysis provides. The height sensor is a signal from the piezoelectric Z position sensor. Usually, a topography image is presented as a height map, but it does not always support the correct picture of the object. More often, creating a pseudo-3D image from the height data makes it easier to interpret. Sometimes, however, the amplitude image gives a clear picture as it is the equivalent to the map of the slope of the object. However, the amplitude scale is inadequate to the object structure as it shows how the tip deflects when it encounters a given topography. The best amplitude image is obtained when its signals are minimized because the amplitude images are error signals from the feedback loop trying to keep the cantilever constant above the surface topography. The phase images, available in tapping mode, are the map of how the phase of cantilever oscillation is affected by its interaction with the surface. The physical meaning of this signal is complicated, as in addition to topographic information, the phase can be affected by the relative softness/hardness of the investigated object or its chemical nature. Overall, it is easy to obtain phase contrast in mixed (nonhomogeneous) objects.^67^ Therefore, the types of images that best reflect the surfaces with a given texture were chosen (Figure 8).

Figure 8A,B demonstrates a nonmesogenic thermoplastic initial PLLA film with a molecular disorder, while Figure 8C shows a nematic bulk mesophase arrangement with a long-range molecular order^6^ of the pressed PLLA film obtained at a pressure of 5 tons for 1 min at 110 °C with a thread-like texture. Large-scale AFM images (20 × 20 μm, Figure 8J) revealed an agglomeration of fibrils forming longitudinal rod-like patterns, which are the dominant feature of the LCP surface.^65^

LC structures are made up of rigid (mesogens) and flexible parts. The rigid part aligns the molecules in a certain direction, while the flexible part induces fluidity into the LC.^5,68^ For pressed PLLA films obtained with a processing temperature close to the *T*_m_ of the polymer, areas of clear phase separation between the rigid mesogens and the relatively soft part of the polymer were also observed, which is more obvious in the phase image, which gives better contrast for heterogeneous objects (Figure 8C/iii). Such phase separation was also observed for the colored planar texture obtained for 2 min at 100 °C (Figure 8D). The AFM study of the surface of the pressed PLLA films with colored planar texture revealed, similarly to SEM, oily streak texture (Figure 8I). Figure 8E,F shows a typical helical orientational order of the rod-like mesogens of a chiral nematic arrangement without surface alignment of the layers. In the case of the BPIII* of the pressed PLLA film (Figure 8G,H), the crystallographic orientation of the BP resembles a staircase pattern.^69^

## Conclusions

The conducted research shows that the nonmesogenic thermoplastic polymer can exhibit stable LC properties at ambient temperature after inducing this state by special conditions under stresses. Additionally, the chiral nematic mesophase was obtained by appropriate regulation of the temperature, heating time, and pressure force, as well as by adding a fine powder (talc). When the sample was cooled to RT, the polymer retained the structure of the nematic mesophase. DSC studies revealed the presence of the LC mesophase by detecting changes in enthalpy associated with phase transitions. The amount of energy released and absorbed was small, which is usually associated with the nematic mesophase. This mesophase was identified by POM, also with the use of variable temperature. It has been shown that the pressing temperature and time during the preparation of PLLA film have a great influence on the type of texture. Therefore, their thermotropic properties can be easily modulated by temperature and time changes during processing, which means that the possibilities of using these films will expand considerably. The amorphous pressed PLLA film with the observed BP was obtained at a processing temperature from 30 to 60 °C, regardless of the force of the pressure applied and the pressing time. The advantage of the obtained polymer material, in addition to being easy to obtain, is a wider temperature range (Δ*T* ≈ 9 °C) of its stability between isotropic and N* thermotropic phases, which opens up new possibilities for photonic applications.

## Supporting Information Available

Additional POM photomicrographs, DSC traces, and nematic-to-isotropic transitions (from POM) for all tested materials (PDF)
