# Supplementary material for: Poly(l-Lactide) Liquid Crystals with Tailor-Made Properties Toward a Specific Nematic Mesophase Texture
Source: ACS Sustain Chem Eng. 2022 Mar 2;10(10):3323–34. doi: 10.1021/acssuschemeng.1c08282 (PMC8924921; doi:10.1021/acssuschemeng.1c08282)
Supplement: Supplementary file 1 — sc1c08282_si_001.pdf [file sc1c08282_si_001.pdf]

Supporting Information

Poly(*L*-lactide) Liquid Crystals with Tailor-Made Properties Towards a Specific Nematic Mesophase Texture

Henryk Janeczek[§], Khadar Duale[§], Wanda Sikorska[§], Marcin Godzierz[§], Aleksandra Kordyka[§], Andrzej Marcinkowski[§], Anna Hercog[§], Marta Musioł[§], Marek Kowalczuk[§,#], Darinka Christova[‡], and Joanna Rydz[§]*

[§]Centre of Polymer and Carbon Materials, Polish Academy of Sciences, M. Curie-Skłodowska 34, 41-800 Zabrze, Poland

[#]School of Science, Faculty of Science and Engineering, University of Wolverhampton, Wulfruna St., Wolverhampton WV1 1LY, UK

[‡]Institute of Polymers, Bulgarian Academy of Sciences, Akad. Georgi Bonchev Str., Bl. 103A, 1113 Sofia, Bulgaria

*corresponding author: (J.R.) jrydz@cmpw-pan.edu.pl

Supporting Information contains 11 pages and 21 figures

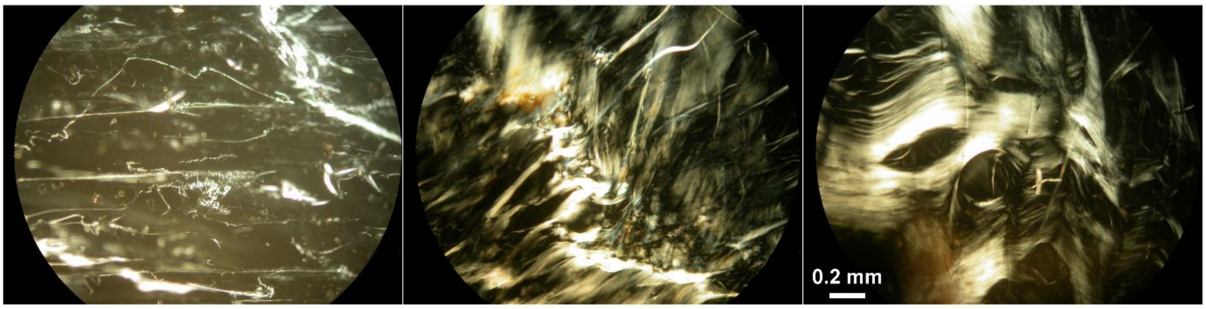

Figure S1. Representative photomicrographs of the thread-like optical texture of the nematic mesophase from several places of pressed PLLA films obtained after pressing at a pressure of 5 tons for 1 min at 110 °C (crossed polarizers, 25 °C, 100X).

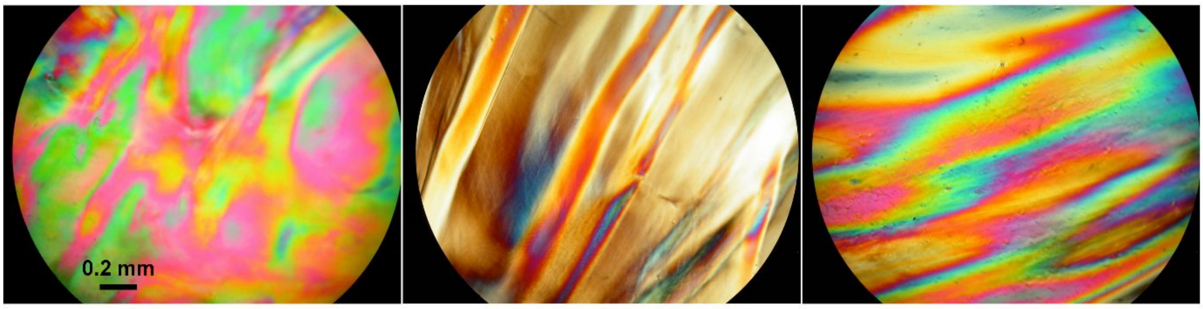

Figure S2. Representative photomicrographs of optical textures of the chiral nematic mesophase from several places of pressed PLLA/talc films with 0.5 wt% of talc obtained after pressing at a pressure of 5 tons for 1 min at 110 °C (crossed polarizers, 25 °C, 100X).

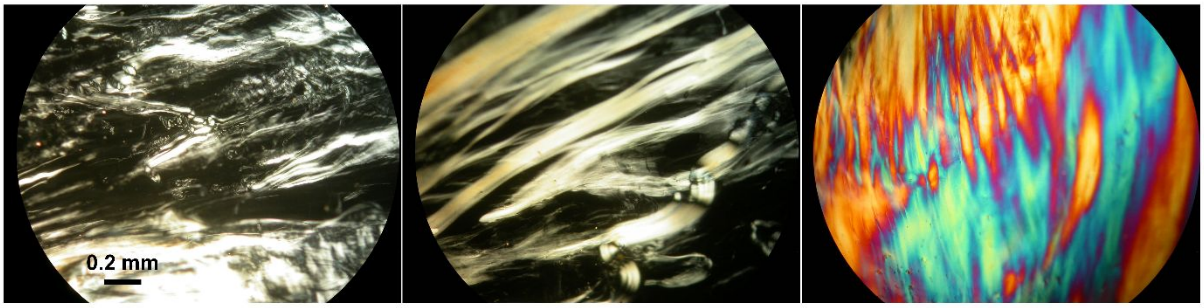

Figure S3. Representative photomicrographs of optical textures of the nematic mesophase with a heterogeneous surface from several places of pressed PLLA/talc films with 0.1 wt% of talc obtained after pressing at a pressure of 5 tons for 1 min at 110 °C (crossed polarizers, 25 °C, 100X).

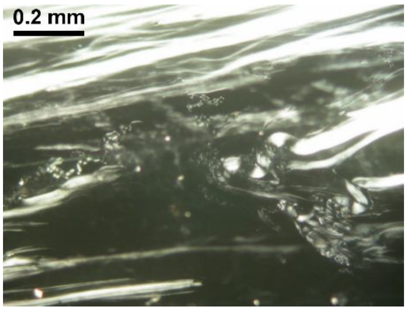

Figure S4. Photomicrograph of optical textures of the schlieren texture (enlarged 100X image showing topological defects) of nematic mesophase of the pressed PLLA/talc film with 0.1 wt% of talc obtained after pressing at a pressure of 5 tons for 1 min at 110 °C (crossed polarizers, 25 °C).

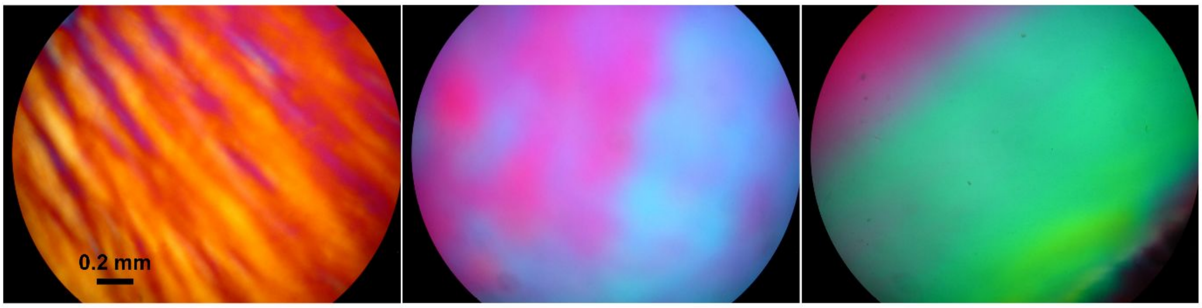

Figure S5. Representative photomicrographs of the blue phase texture from several places of pressed PLLA films obtained after pressing at a pressure of 5 tons for 1 min at 40–60 °C (crossed polarizers, 25 °C, 100X).

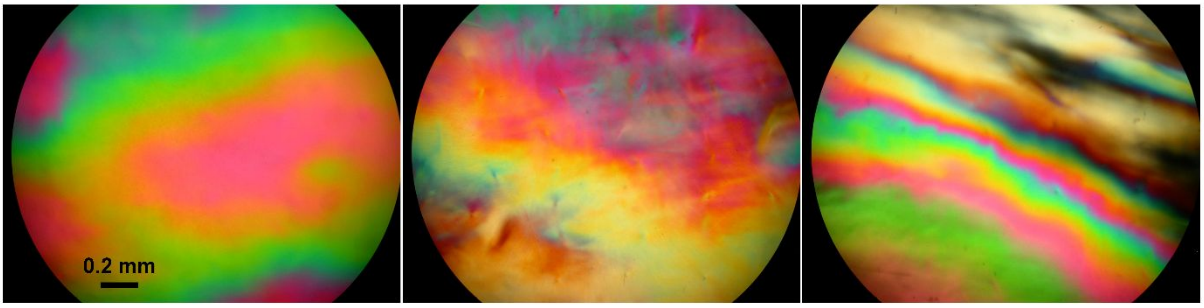

Figure S6. Representative photomicrographs of the color fog texture from several places of pressed PLLA films obtained after pressing at a pressure of 5 tons for 1 min at 70 °C (crossed polarizers, 25 °C, 100X).

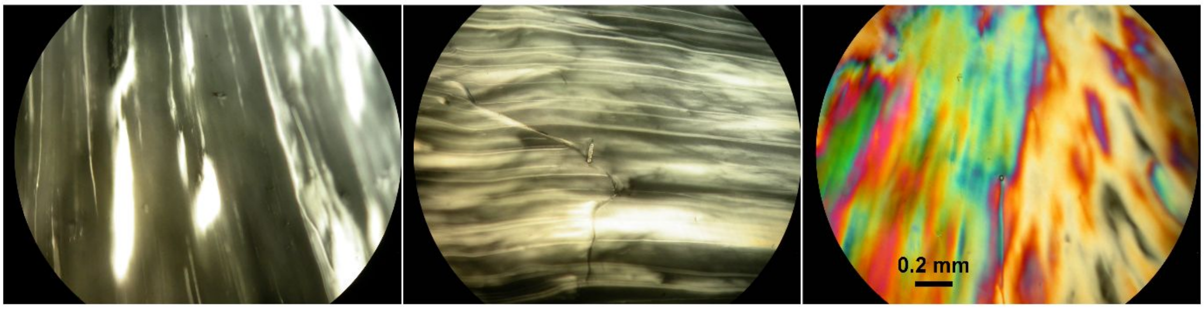

Figure S7. Representative photomicrographs of optical textures of the nematic mesophase with a heterogeneous surface from several places of pressed PLLA films obtained after pressing at a pressure of 5 tons for 1 min at 80 °C (crossed polarizers, 25 °C, 100X).

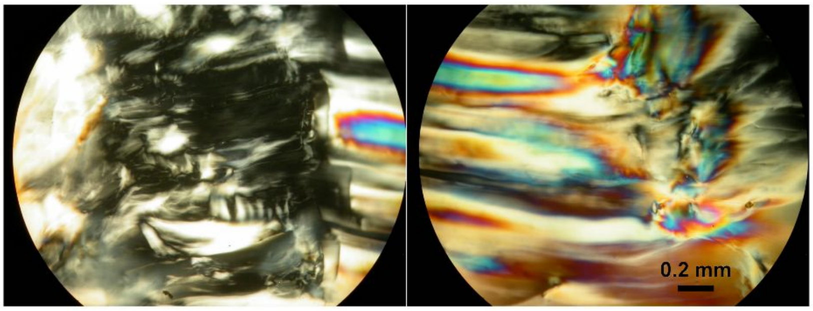

Figure S8. Representative photomicrographs of optical textures of the nematic mesophase with a heterogeneous surface from several places of pressed PLLA films obtained after pressing at a pressure of press jaws for 2 min at 80 °C (crossed polarizers, 25 °C, 100X).

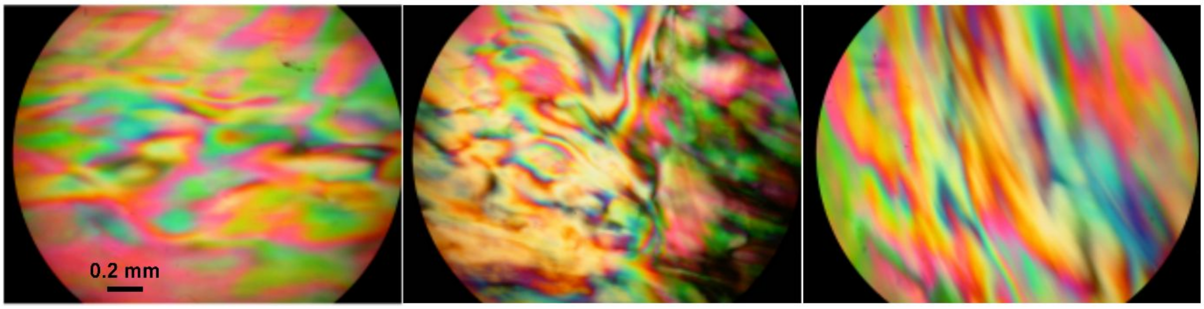

Figure S9. Representative photomicrographs of optical textures of the colored planar Grandjean texture of the chiral nematic mesophase from several places of pressed PLLA films obtained after pressing at a pressure of 5 tons for 2 min at 80 °C (crossed polarizers, 25 °C, 100X).

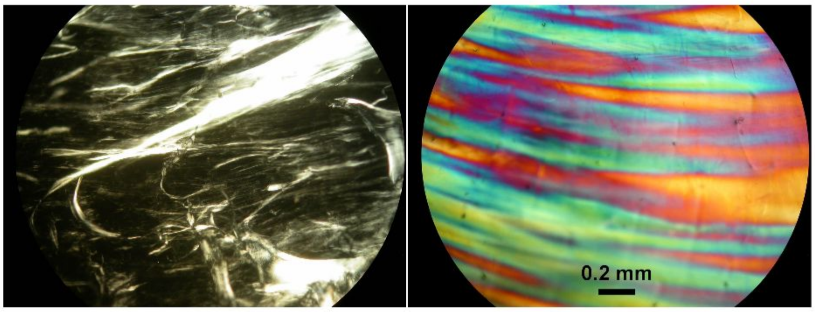

Figure S10. Representative photomicrographs of optical textures of the nematic mesophase with a heterogeneous surface from several places of pressed PLLA films obtained after pressing at a pressure of 5 tons for 2 min at 110 °C (crossed polarizers, 25 °C, 100X).

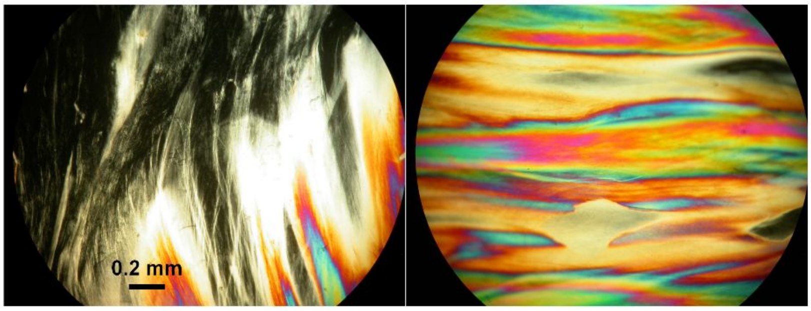

Figure **S11.** Representative photomicrographs of optical textures of the nematic mesophase with a heterogeneous surface from several places of pressed PLLA films obtained after pressing at a pressure of 5 tons for 3 min at 110 °C (crossed polarizers, 25 °C, 100X).

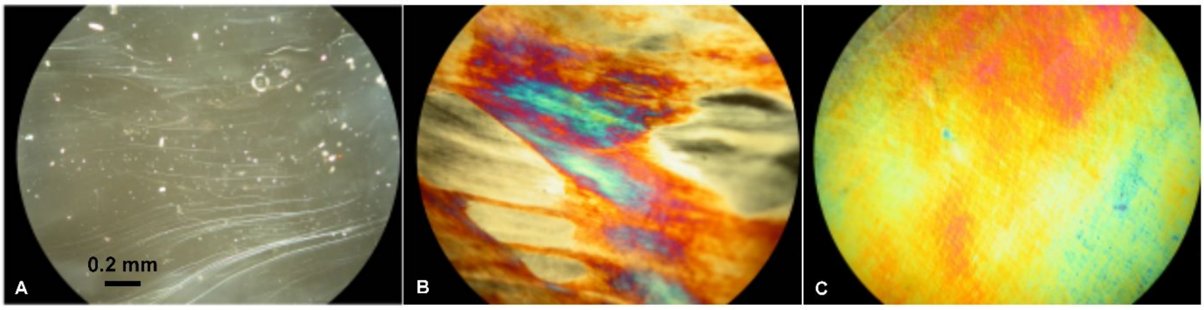

Figure S12. Representative photomicrographs of optical textures of the nematic mesophase from several places of the other pressed PLLA films (starting PLLA obtained by solution casting method) obtained after pressing at a pressure of 5 tons for 1 min at 110 °C (thread-like texture, A), for 2 min at 80 °C (colored planar texture of the chiral nematic mesophase, B), and at 50 °C (blue phase texture, D) (crossed polarizers, 25 °C, 100X).

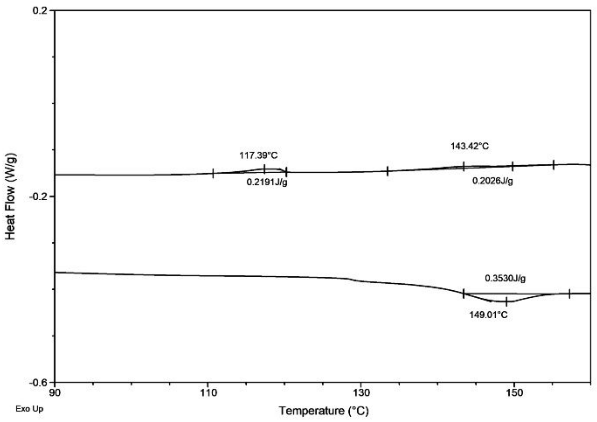

Figure S13. DSC traces of the pressed PLLA film (from the starting PLLA film obtained by solution casting method) with a colored planar texture of the chiral nematic mesophase gained after pressing at a pressure of 5 tons for 2 min at 80 °C; DSC obtained at 10 °C·min^{-1} in the cooling run and second heating run.

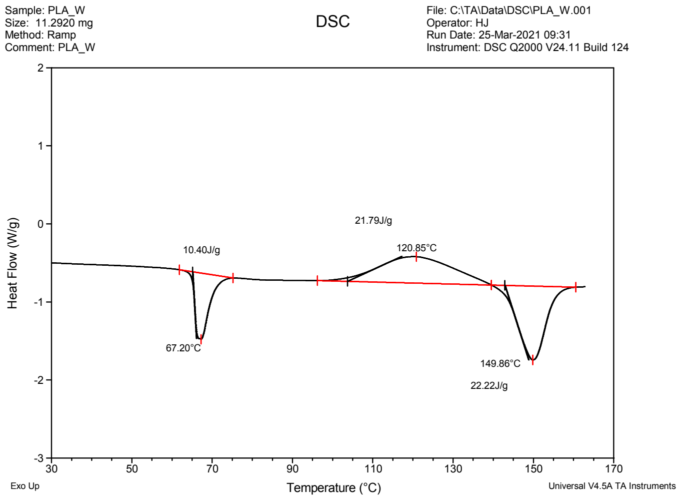

A

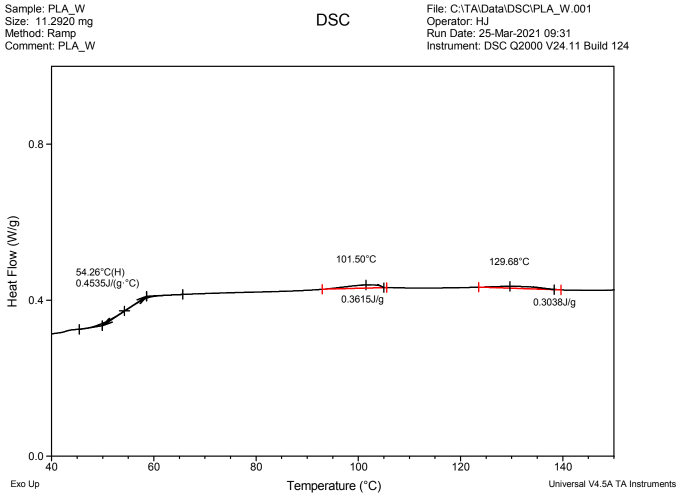

B

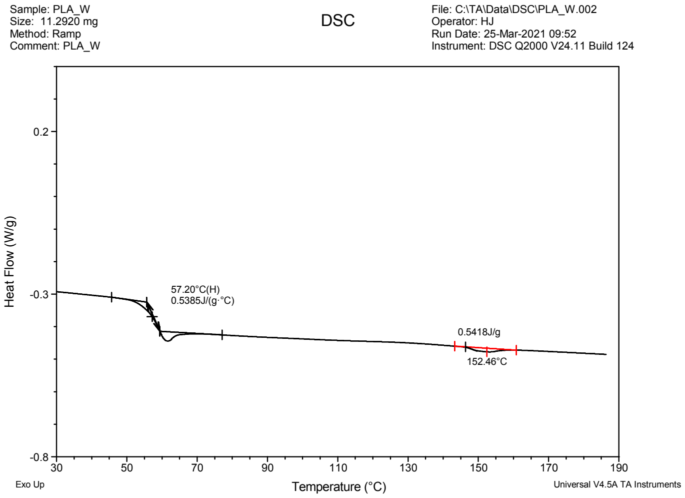

C

Figure S14. Original DSC traces of the initial PLLA rigid film; DSC obtained at 20 °C·min⁻¹ in the first heating run (**A**), at 10 °C·min⁻¹ in the cooling run (**B**), and at 10 °C·min⁻¹ in the second heating run (**C**).

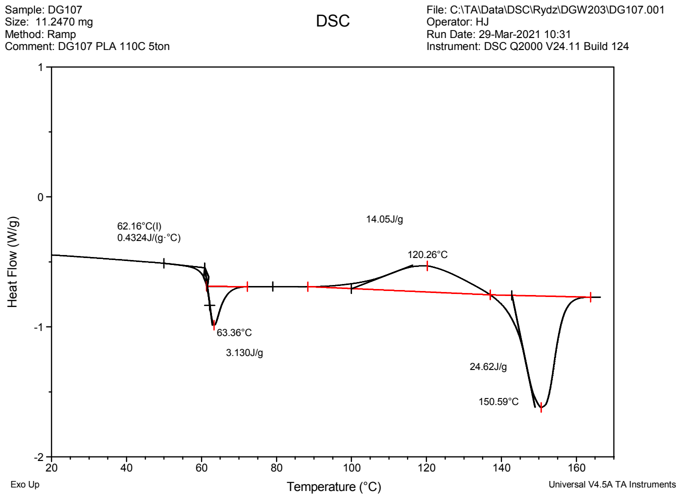

A

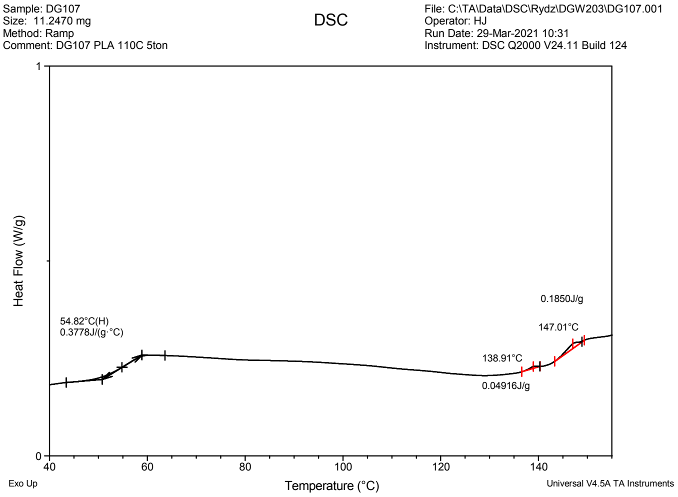

B

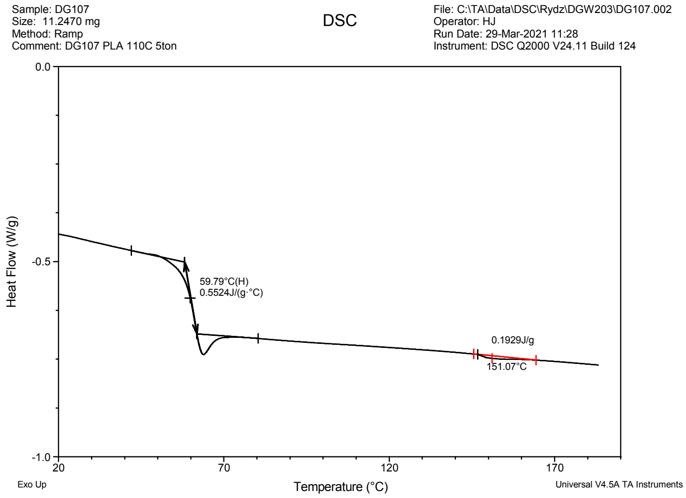

C

Figure S15. Original DSC traces of the pressed PLLA films obtained at a pressure of 5 tons for 1 min at 110 °C (thread-like texture); DSC obtained at 20 °C·min⁻¹ in the first heating run (A), at 10 °C·min⁻¹ in the cooling run (**B**), and at 10 °C·min⁻¹ in the second heating run (**C**).

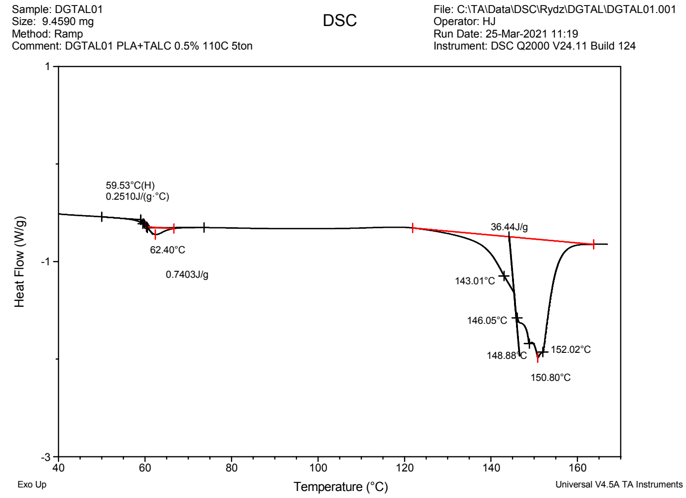

A

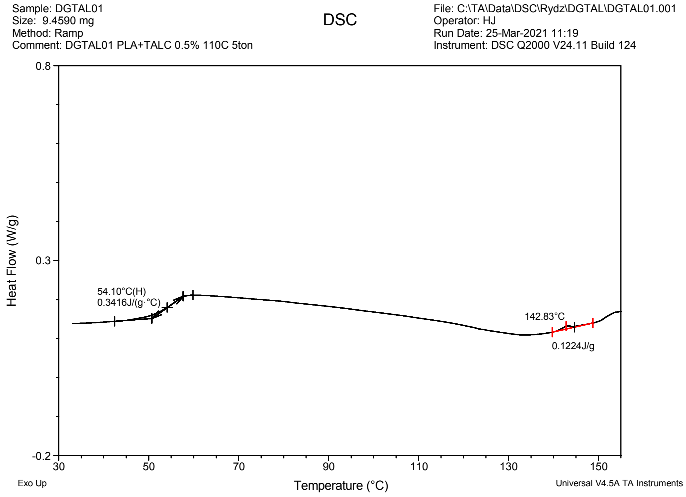

B

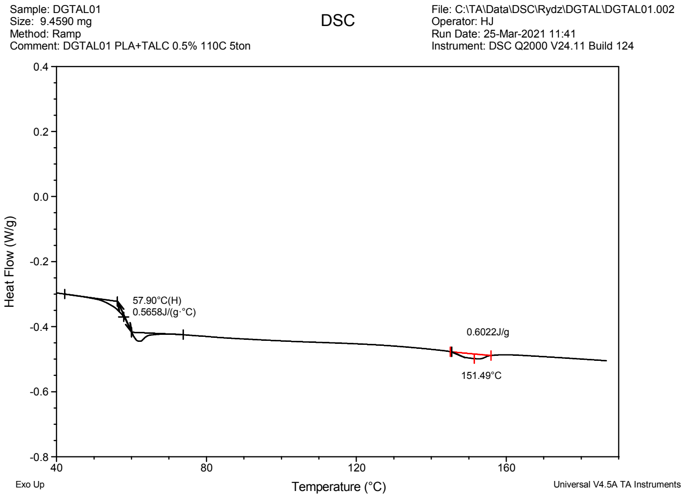

C

Figure S16. Original DSC traces of the pressed PLLA/talc film with 0.5 wt% of talc obtained at a pressure of 5 tons for 1 min at 110 °C; DSC obtained at 20 °C·min^{-1} in the first heating run (A), at 10 °C·min^{-1} in the cooling run (**B**), and at 10 °C·min^{-1} in the second heating run (**C**).

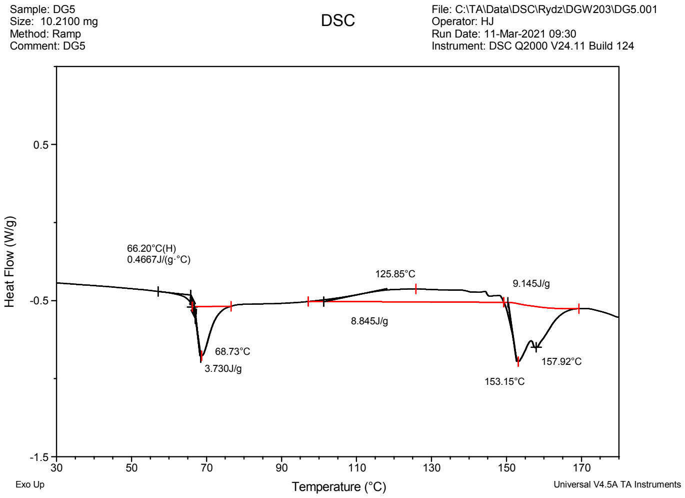

A

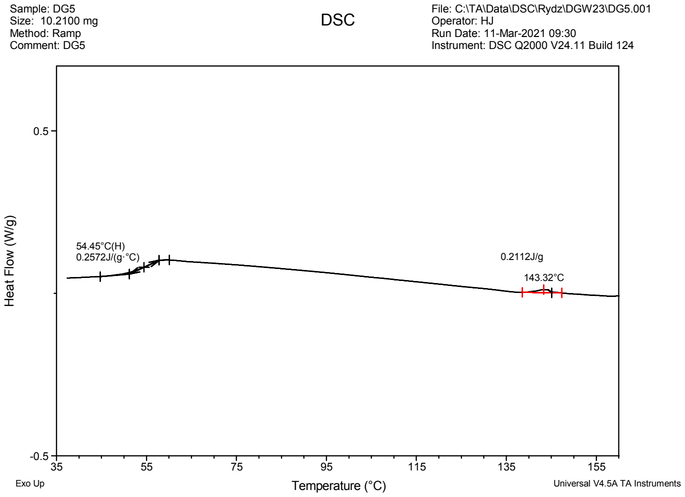

B

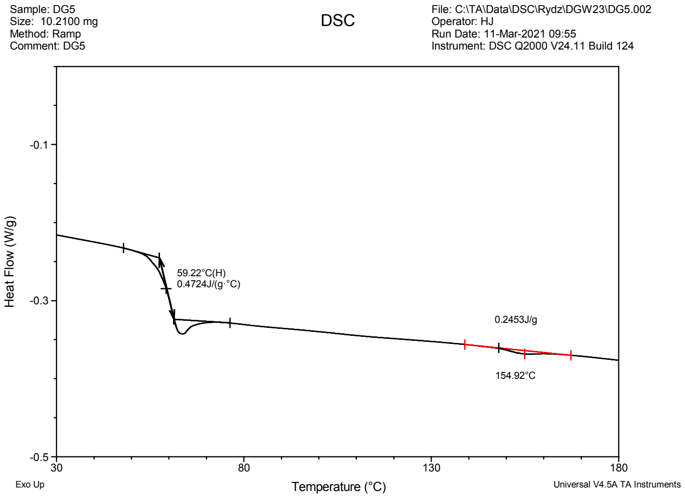

C

Figure S17. Original DSC traces of the pressed PLLA films obtained at a pressure of 5 tons for 2 min at 100 °C (colored planar texture); DSC obtained at 20 °C·min⁻¹ in the first heating run (A), at 10 °C·min⁻¹ in the cooling run (**B**), and at 10 °C·min⁻¹ in the second heating run (**C**).

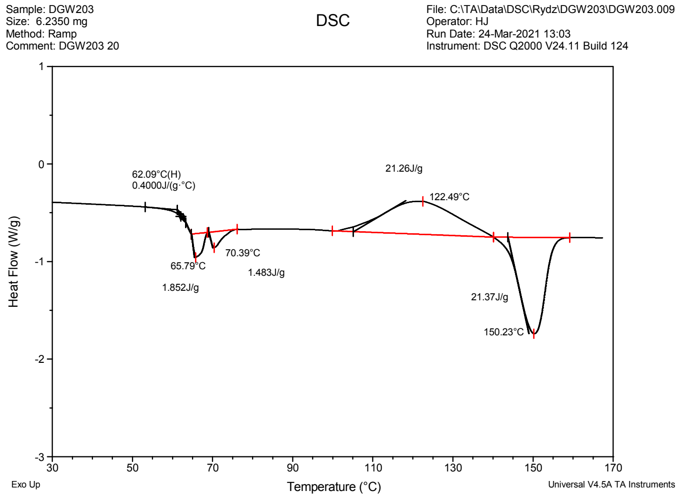

A

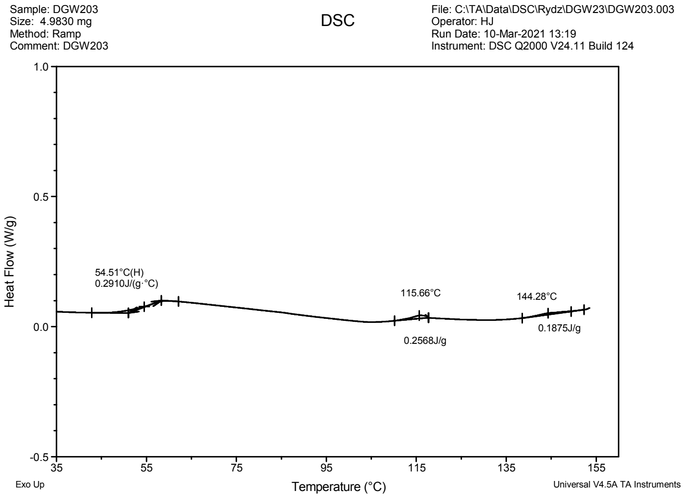

B

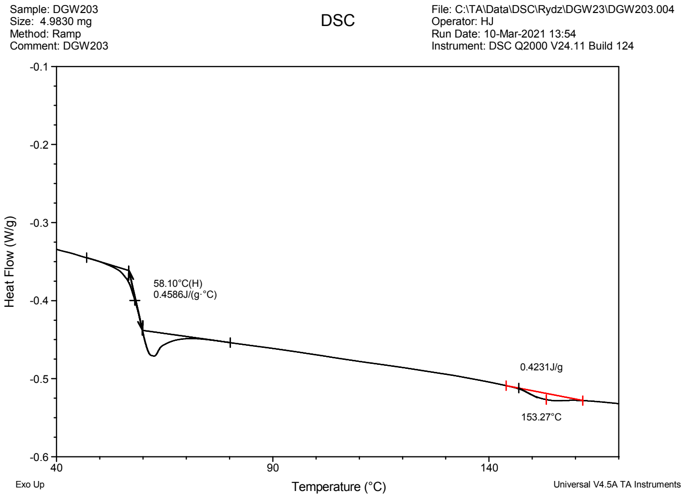

C

Figure S18. Original DSC traces of the pressed PLLA films obtained at a pressure of 5 tons for 1 min at 50 °C (BPIII*); DSC obtained at 20 °C·min^{-1} in the first heating run (A), at 10 °C·min^{-1} in the cooling run (**B**), and at 10 °C·min^{-1} in the second heating run (**C**).

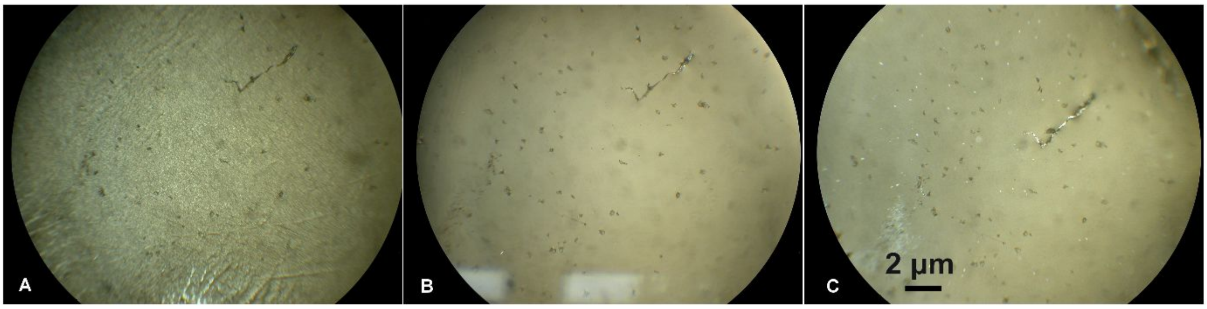

Figure S19. Photomicrographs of the optical texture of amorphous initial PLLA film during heating and cooling at 154 °C (A), 158 °C (isotropic phase, B) and 95 °C (amorphous with slow crystallization during cooling, C) (crossed polarizers, 160X).

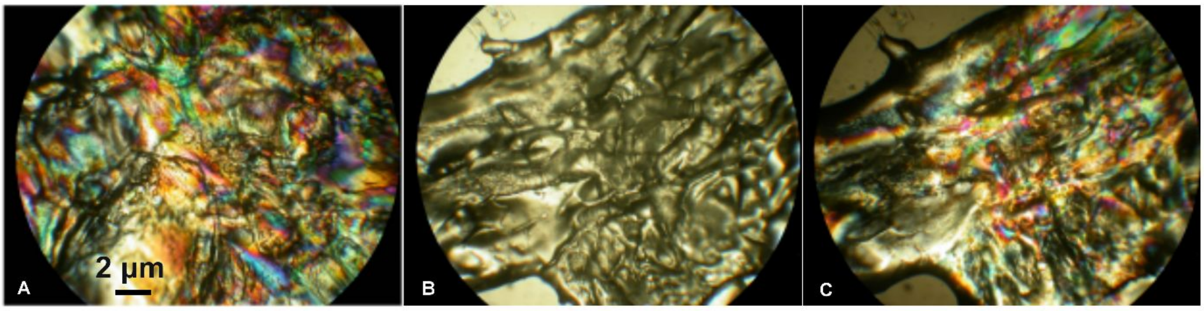

Figure S20. Reversible nematic to isotropic transition of the pressed PLLA/talc with 0.5 wt%. Photomicrographs of the optical texture of the chiral nematic enantiotropic mesophase during heating and cooling at 144 °C (nematic mesophase, A), 154 °C (isotropic phase, B) and 145 °C (nematic mesophase after cooling, C) (crossed polarizers, 160X).

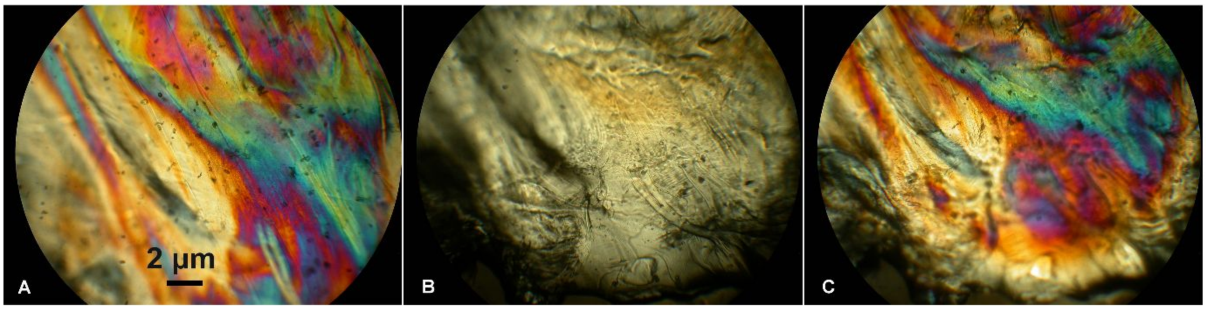

Figure S21. Reversible nematic to isotropic transition of the pressed PLLA with colored planar texture. Photomicrographs of the optical texture of the chiral nematic enantiotropic mesophase during heating and cooling at 140 °C (nematic mesophase, A), 152 °C (isotropic phase, B) and 140 °C (nematic mesophase after cooling, C) (crossed polarizers, 160X).
